# Resilience in Plant-Herbivore Networks during Secondary Succession

**DOI:** 10.1371/journal.pone.0053009

**Published:** 2012-12-27

**Authors:** Edith Villa-Galaviz, Karina Boege, Ek del-Val

**Affiliations:** 1 Centro de Investigaciones en Ecosistemas, Universidad Nacional Autónoma de México, Morelia, Michoacán, México; 2 Instituto de Ecología, Universidad Nacional Autónoma de México, México D. F., México; National Institute of Water & Atmospheric Research, New Zealand

## Abstract

Extensive land-use change in the tropics has produced a mosaic of successional forests within an agricultural and cattle-pasture matrix. Post-disturbance biodiversity assessments have found that regeneration speed depends upon propagule availability and the intensity and duration of disturbance. However, reestablishment of species interactions is still poorly understood and this limits our understanding of the anthropogenic impacts upon ecosystem resilience. This is the first investigation that evaluates plant-herbivore interaction networks during secondary succession. In particular we investigated succession in a Mexican tropical dry forest using data of caterpillar associations with plants during 2007–2010. Plant-herbivore networks showed high resilience. We found no differences in most network descriptors between secondary and mature forest and only recently abandoned fields were found to be different. No significant nestedness or modularity network structure was found. Plant-herbivore network properties appear to quickly reestablish after perturbation, despite differences in species richness and composition. This study provides some valuable guidelines for the implement of restoration efforts that can enhance ecological processes such as the interaction between plants and their herbivores.

## Introduction

At the global scale, a large proportion of land has been modified for agriculture and cattle production (21.8% transformation worldwide) [1]. The subsequent abandonment of this land following soil depletion is a common practice, and gives rise to a mosaic of secondary forests of varying successional status. One of the most threatened ecosystems is the tropical dry forest (TDF) [1], [2]. In Latin America, 66% of the original land cover of this ecosystem has been lost [3] and in Mexico 73% of the original TDF have disappeared [2], highlighting the conservation relevance of secondary forests left [4], [5].

During secondary succession, resource availability varies along the successional gradient, influencing species population dynamics as well as community structure and diversity at all trophic levels [6]. Most studies of the effects of secondary succession on biodiversity have concentrated on plant communities, particularly those of the TDF (but see Chazdon et al. 2011 [7])and therefore our understanding of forests succession for the other components of this ecosystem and its species interactions remains poorly known [8], [9].

Lack of information regarding the different biotic components across succession in tropical ecosystems [10], together with the difficulty of extrapolating information of successional studies in different ecosystems [11], limit our ability to predict different scenarios of deforestation and land use change in TDF, for the purposes of biodiversity conservation [4], [8], [9]. Biotic interactions may respond differently to land use changes in TDF compared to other ecosystems, due to the high annual climatic variability and the presence of seasonal climatic extremes. In TDF, plants lose their foliage during the dry season (6 to 8 months per year), leaving insects without food [9] and impelling specific evolutive strategies in the herbivores. Colonization and establishment of plant propagules is often challenged by the long dry season, during which small seedlings face extreme drought [12].

In order to study the repercussions of land-use change for the biota and the process of community recovery and restoration, we must go beyond the commonly used community descriptors of species richness and diversity as these fail to consider species interactions and ecosystem functioning [13]. How fast do ecological processes recover after anthropogenic perturbation? A useful tool with which to address this question is the assessment of ecological networks to evaluate and predict of the consequences of ecosystem perturbation on the characteristics of ecological processes such as the interaction among species [14].

The structure and topology of ecological networks affects community response to perturbations in mutualistic and antagonistic networks [15]. For example, some intrinsic characteristics observed in ecological networks, such as high nestedness and modularity, confer a greater resilience against species extinction [16], [17] and allow the development of ecological indices with which to evaluate global network resilience [18], [19]. Recently Passmore *et al.* [20] investigated the response of a ant-plant mutualistic network in the Amazonian forest fragments and found high resilience suggesting that networks can resist biotic and abiotic consequences of fragmentation.

Despite this useful feature, ecological networks have not been widely utilized in applied ecological studies where they have great potential value addressing questions concerning the restoration of community structure and function [21]. This is the first investigation that evaluates the utility of ecological networks parameters as an indicator of ecological processes recovery after anthropogenic perturbation, in particular assessing the resilience of plant-herbivore interactions along a secondary successional gradient in a TDF. We demonstrate that plant-caterpillar networks recover their properties very quickly during the natural regeneration of TDF, and that the secondary succession stages and mature forest networks are similar in terms of the ecological network structure descriptors, although some differences in lepidopterous community composition were found.

## Materials and Methods

### Study site

This study was carried in the Chamela-Cuixmala Biosphere Reserve (CCBR, 19°22′–19°39′N, 104°56′–105°10′W) and its surroundings, in Jalisco, Mexico. Average annual rainfall is 748 mm but varies greatly from year to year (from 453 to 1393 mm), and is concentrated (80%) within a rainy season from July to October [22]. The vegetation within the 13,142 hectares of the reserve consists primarily of tropical dry forest (1149 plant species with an average canopy height of 6 m), and semi-deciduous forest established along the larger streams (average canopy height of 10 m) [23]. The TDF found at Chamela-Cuixmala is considered one of the most diverse of its kind, comprising a high percentage of endemic plant species [2], [23]. The invertebrate inventory is quite small; however, 1877 species have been described in the reserve, 583 of which are lepidopteran species [24].

Land use around the reserve has been transformed by human activity, mainly related to agriculture and livestock production, leading to 16–17% deforestation in the buffer zone. Consequently, the landscape of the region is now a mosaic of intact original vegetation, agricultural fields and secondary forests at different stages of succession [25].

### Experimental design

The study sites are integrated into the experimental design of the CIECO-UNAM Tropical Forest Management project (MABOTRO) which features a successional chronosequence of abandoned agricultural sites [26]. This chronosequence is formed by four stages of secondary succession: Initial (fields abandoned for four years), early secondary forest (6–9 years of abandonment), late secondary forest (13–16 years of abandonment) and mature forest. There were three replicates of each successional stage with the exception of the initial stage, which only had two due to a fire in one location. Each replicate consisted in 20×50 m plots, with four transects of 2×20 m established every 10 m within each plot. Excluding lianas, all woody plants ≥1 cm in diameter and ≥50 cm within these transects were labeled. Plot size was 1 ha at least (mature forests are immersed in the biosphere reserve therefore they are greater in size) and the distance between plots of the same successional stage was 3 km.

During the rainy season of each year in the period 2007–2010, surveys were carried out in labeled plants searching for caterpillars on all leaves and stems. A total of 19 surveys were conducted. In order to search for caterpillars on adult trees, a subsample of three branches - comprising approximately 100 leaves - was taken per individual tree. Caterpillars were recorded and raised in the laboratory to procure adults for subsequent taxonomic identification. Plant material was also collected and identified to at least family level.

### Network topology

For each successional stage replicate, a bipartite network was constructed and the following network structure descriptors were calculated: network size, connectance, nestedness, extinction curve, robustness, number of compartments, network specialization and modularity, the data from all samplings was incorporated to create a single network per plot. 1) Network size was defined as the total richness of species, 2) connectance (*C*) as the fraction of recorded interactions relative to the total possible interactions [27], 3) nestedness (*N*) is a topological pattern in which the majority of the specialist species interact exclusively with more generalist species that also interact with them in return [28]. This descriptor was calculated with the nested overlap and decreasing fill (NODF) metric [29] using Aninhado 3.0 software [30], while the significance was estimated with a Monte Carlo procedure, performing 1000 randomizations created from the null model *Ce*, in which the probability of an interaction between an animal and a plant is proportional to the total observed number of their interactions. The measure itself is unsuitable for drawing comparisons between different networks due to the fact that it is affected by network size and connectance [28]. Thus, the relative nestedness (N*) is defined as
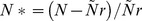
where *N* is the value of nestedness of the current matrix while *Ñr* refers to the average value of nestedness of the random replicates. 4) Number of compartments: these are network subgroups that are not connected to another subgroup [13], related to 5) the modularity (*M*) that measures the level at which the species tend to be organized in subgroups of species that interact more frequently among themselves than with other members of the network [17]. This value was calculated with the index *M* (range 0–1) in the program NETCARTO [31], based on the algorithm of Newman & Girvan [32], while its significance was estimated using a Monte Carlo procedure of 1000 randomizations. Random matrices were generated with the *vaznull* function of the bipartite package of the program R [33], and were generated from the characteristics of the networks of each plot of each successional stage. This function is based on the algorithm of Vazquez *et al.* [34], and observes both the marginal totals as well as the connectance of the matrix. This latter criterion represents the ecological restrictions of the network interactions [35]. To carry out comparisons between networks, relative modularity (M*) was calculated as
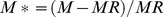
where *M* is the value of nestedness of the current matrix, while MR refers to the average value of modularity of the random replicates.

To evaluate changes in network specialization, the metric *H_2_*′ was calculated [36]. This is a measure of specialization at the network level that is based on the deviation between the number of interactions of a species and the expected total of interactions for each network, assuming that all species interact with their partners in proportion to their observed frequency totals. This metric has the advantage that it is not affected by sampling effort, network size or symmetry, allowing robust and reliable comparisons among networks [36].

Similarly, robustness was calculated to evaluate vulnerability to extinction in the lepidopterous community within each successional stage [19]. This descriptor refers to the area below the extinction curve [18] created by the fraction of species that survive following the removal of a given species, calculated as the exponent *a* of the function *y = 1-x^a^*. Extinctions were carried out by randomly removing plants from the network. When a lepidopterous species was connected exclusively to the removed species, it was also removed from the network. In this way, an extinction curve was generated by plotting the number of species that remained within the network against the accumulated number of removed species (1000 randomizations).

Linear mixed effect models were used in order to determine whether there were differences between the network parameters in each different successional stage [37]. For these models, the successional stage was the fixed effect while the years of study within the same plot were the random effects. In all cases, successional stage was the independent variable. For the measurements of connectance, extinction curve and robustness, network size was also used as a cofactor. This was because of the correlation between this factor and the other descriptors. Number of compartments was incorporated as a cofactor in the connectance model.

## Results

**Figure 1:**
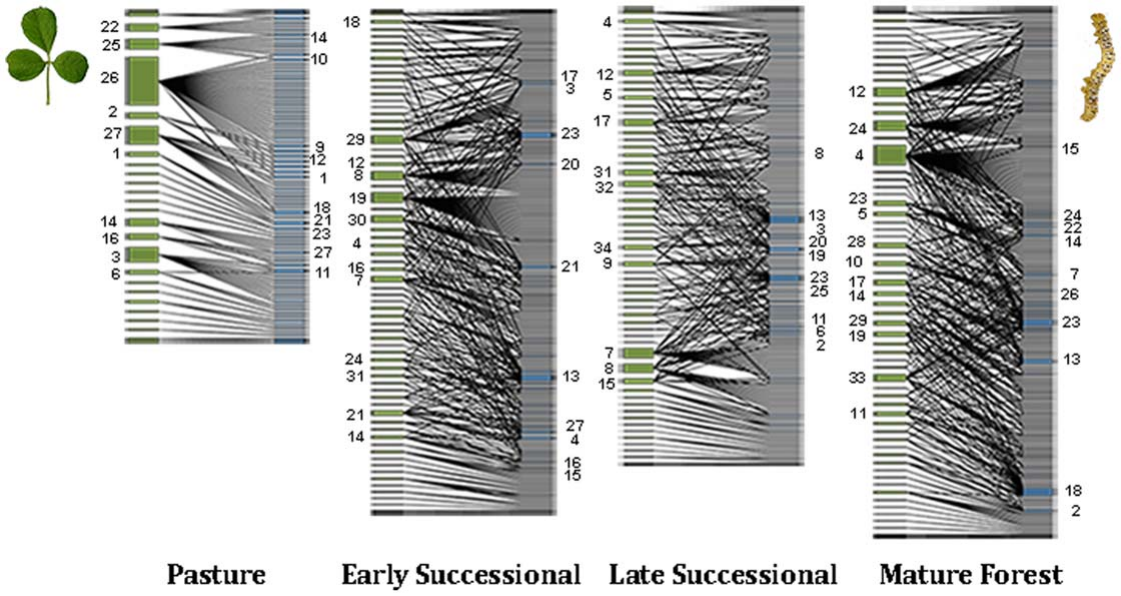
Plant-herbivore networks in different successional stages. Each network represents all the interactions observed in all the sampling times and in all replicates of the same successional stage. Species bar size represents the number of interactions it has. **Plants are represented in green**: 1) *Acacia farnesiana*, 2) *Acacia macracantha*, 3) *Lonchocarpus* sp. L, 4) *Apoplanesia paniculata*, 5) *Ayenia micrantha*, 6) *Coccoloba liebmannii*, 7) *Caesalpinia caladenia*, 8) *Casearia nitida*, 9) *Colubrina triflora*, 10) *Coursetia caribaea*, 11) *Croton pseudoniveus*, 12) *Dalbergia congestiflora*, 13) *Guapira macrocarpa*, 14) *Gyrocarpus jatrophifolius*, 15) *Heliocarpus pallidus*, 16) *Hintonia latiflora*, 17) *Justicia candicans*, 18) *Leucaena lanceolata*, 19) *Lonchocarpus* sp. 2, 20) *Lonchocarpus eriocarinalis*, 21) *Lonchocarpus* sp. F, 22) *Lonchocarpus* sp. A, 23) *Lonchocarpus* sp. K, 24) *Mimosa arenosa*, 25) *Mimosa pigra*, 26) *Phyllanthus mocinianus*, 27) *Piptadenia constricta*, 28) *Rauvolfia tetraphylla*, 29) *Spondias purpurea*, 30) *Stemmadenia donnell-smithii*, 31) *Thouinia paucidentata*, 32) *Trichilia trifolia*. **Lepidopterans are represented in blue**: 1) *Agraulis vanillae incarnate, 2) Anomis editrix, 3)Apatelodes pudefacta, 4) Automeris io, 5)Dasylophia eminens, 6) Eutelia auratrix, 7) Geometridae sp. 10, 8) Geometridae sp. 12, 9) Geometridae sp. 7, 10) Geometridae sp. 9, 11) Gonodonta pyrgo, 12) Norape tenera, 13) Orgya sp., 14) Polygonus manueli manueli, 15) Syllepsis hortalis, 16) Wockia chewbacca, 17) Arctiidae sp. 1, 18) O114, 19)O14, 20) O18, 21)O190, 22 Geometridae sp. 34, 23)O3, 24)O32, 25) Geometridae sp. 59, 26)O67, 27)O80*.

We found 140 tree species (Appendix S1) interacting with 471 Lepidopteran species along secondary succession (Appendix S2). The size of the plant-caterpillar network differed between successional stages (Fig. 1). The initial succession network in particular was significantly smaller (y¯ = 18±3.62, standard deviations from hereafter) than the other successional stages (y¯ = 46.08±2.8; *F*_3,37_ = 19.71, *p* = 8.43 e^−08^). Number of compartments also differed between successional stages, with the pasture networks presenting an average of 4.12±0.24 compartments for the initial successional stage in comparison to 7.94±0.18 in the later stages (F_3,36_ = 5.573, *p* = 0.00019). Connectance of the interaction network presented the same pattern, grouping the early and late succession stages together with the mature forest (y¯ = 0.08±0.003 which differed from the initial stage that had greater connectance (y¯ = 0.22±0.021; F_3, 36_ = 51.477, *p* = 4.21 e^−13^; Tables 1 and 2). Robustness was different between stages (F_3,36_ = 3.666, *p* = 0.02104), given that robustness in the initial stage was lower than than in older stages (y¯ = 0.53±0.041 [see Table 1 and 2]).

**Table 1:** Network measurements in four successional stages (Mean ± SD) of the tropical dry forest in the Chamela region.

*Network descriptor*	*Pasture*	*Initial*	*Intermediate*	*Mature*	*F value*	*p*
***Network size***	18±3.61	45.9±7.16	41.34±3.37	51±4.96	F_3, 37_ = 19.71	**8.43 e^−08^**
***Connectance***	0.22±0.02	0.085±0.009	0.09±0.009	0.08±0.004	F_3, 36_ = 51.47	**4.21 e^−13^**
***Nestedness***	0.575 0.0344	0.364±0.08	0.329±0.058	0.33±0.052	--------	ns
***Modularity***	0.044±0.09	0.18±0.056	0.31±0.12	0.15±0.032	-------	ns
***Number of compartments***	4.125±0.24	8.166±0.75	8.08±1.32	7.58±0.64	F_3, 36_ = 5.573	**1.9 e^−04^**
***Extinction curve***	1.2±0.20	1.44±0.043	1.52±0.05	1.57±0.12	F_3, 36_ = 2.066	0.13
***Robustness***	0.53±0.04	0.6±0.02	0.6±0.009	0.56±0.043	F_3, 36_ = 3.666	**0.021**
***Network specialization***	0.81±0.02	0.74±0.06	0.792±0.05	0.735±0.061	F_3_ = 0.55	0.648
***Caterpillars (no. spp)***	12.25±3.04	29.75±5.9	26.25±2.44	33.58±4.13	F_(3,8)_ = 6.07	0.02
***Plants (no. spp)***	5.75±0 .63	16.17±1.5	15.08±0.956	17.41±0.88	F_(3,8)_ = 27.29	0.0001

**Table 2:** Significant *a posteriori* contrasts.

Descriptor	Model	Contrast	*t* values	*p*
**Network size**	**lme**	Mature forest-pasture	t_7_ = 5.40	p = 0.001
		Late-pasture	t_7_ = 4.33	p = 0.0034
		Early-pasture	t_7_ = 4.841	p = 0.0019
**Connectance**	**lme**	Mature forest-pasture	t_7_ = −5.068	p = 0.0015
		Late-pasture	t_7_ = −6.036	p = 0.0005
		Early-pasture	t_7_ = −8.19	p = 0.0001
**Robustness**	**lme**	Mature forest-pasture	t_7_ = −3.28	p = 0.0135
		Late-pasture	t_7_ = −5.8	p = 0.0008
		Early-pasture	t_7_ = −5.6	p = 0.0006
**Number of compartments**	**lme**	Mature forest-pasture	t_7_ = −4.84	p = 0.0019
		Late-pasture	t_7_ = −4.75	p = 0.0021
		Early-pasture	t_7_ = −4.279	p = 0.0037

No significant differences were found between the nestedness values (NODF) of the random and studied matrices (*p*>0.05). For this reason, the NODF values of all matrices did not differ significantly from those obtained at random. Notably, the observed values were very low on average (<15), likewise for the parameter of modularity. Only some plots in certain years had values that were significantly different to those expected by chance, while these remained non-significant for the majority (*p*>0.05). Moreover, no significant differences were observed in the value of specificity of the network (H^2^) between successional stages (F_3,36_ = 0.55, *p* = 0.648).

## Discussion

Considering that the gradual increase in species is intrinsic to any secondary succession process, it is intuitive that the network of biotic interactions should differ in sites featuring different identities and time since perturbation. Although various studies have reported such increase of diversity in plant [38], [39] and herbivore species across forest succession [40], [41], [42], the relevant and novel aspect of this study is the finding of a rapid recovery of network size and properties after a perturbation of TDF. Six to 13 years after abandonment, succession plots had already network properties equivalent to mature forest plots, which emphasize the importance of secondary forest for the conservation of species but also of ecological processes such as herbivory.

Secondary forests in TDF's usually do not appear to differ widely from primary forests in terms of richness and composition of plant [5], [39], [40] and animal species [43]. In the case of the TDF in this area, a recent investigation has demonstrated that, whereas abundance of herbivores was different only at the initial succession stage, species richness, diversity and composition was different across succession [42]. Despite these differences, network size across succession was similar for the last three successional stages, which suggest that the rapid recovery of structure and diversity of vegetation seems to similarly promote a speedy reintegration of caterpillars to the system, as has been observed in other forests [39].

One interesting aspect is how changes in network size during secondary succession can affect the functionality of the system. Ecosystems are capable of regaining function long before completing the recovery of all their structural attributes [38]. Compared to the tropical humid forest, tropical deciduous forests are considered by Murphy & Lugo [44] to be resilient to environmental perturbations because of its relative simplicity and structure. Although there is no consensus regarding this point of view, evidence of early recovery of TDF has been found in terms of structure and plant diversity [7], [39], as well as in the diversity of birds and bats [7] and caterpillars [42], although with significantly different species composition. A recent study in fragmented humid rainforests [20] showed that ant-plant mutualistic networks are resilient to abiotic and biotic changes caused by fragmentation. Accordingly our investigation in a tropical dry forest showed high resilience in plant-herbivore network across succession, given that differences in network size, connectivity and number of compartments between successional stages were significant only between the initial and the older successional stages (Fig. 1).

Connectance in the studied networks was generally low (0.12±0.034), but higher in the initial succession stage relative to older stages and mature forest. Low connectance is characteristic of antagonistic networks, and can be promoted by high competition between herbivores restricting the exchange of interacting factors, or by the arms race evolutionary dynamics between plants and their consumers. Such low connectance has the advantage of conferring a high stability to the network by reducing the propagation of negative effects throughout the network in the case of punctual extinctions [15]. This suggests a greater resilience in later successional stages, compared to initial successional stages. In this context, restoration efforts should provide particular attention to reduce the factors driving local extinctions in early successional stages, which seem to be the most vulnerable to suffer indirect extinction events, as suggested by their high connectance.

Contrary to our expectation, the networks studied did not present a pattern of significant modular layout and values of modularity were lower than those expected in a network of equal size and connectance [34]. This seemingly random pattern could be the result of several factors: a) skewed representatively of the lepidopterous community, given that the present study only included caterpillar species found in trees and shrubs, thus potentially excluding part of the community specialized to feed on lianas or herbaceous plants [45], b) the high turnover of species (70% turnover between different successional stages and between the two years studied [42]), c) high variation in environmental conditions; rainfall is a key factor in the TDF's biodiversity, and as might be expected, varies from year to year depending on the incidence of hurricanes, which would not favor the presence of modules.

It has been suggested that one of the factors causing an increase in species richness of insects during succession is a greater food specificity as forest mature, allowing the coexistence of a greater number of species [41]. However, studies conducted in tropical rainforests indicate that host specificity by herbivores does not change significantly during succession [11]. In the case of tropical rain forests, this can be explained by the abundance of pioneer trees with chemical defenses, which increase host specificity even in the early successional stages, as occurs in humid tropical forest [11]. In the case of tropical dry forests, however, the boundaries between pioneer and mature forest species are less defined and some late succession species can colonize quite soon recently perturbed areas, whereas pioneer species can be also found in mature forest [7]. This should promote, in turn, a higher range of host species occurrence across forest succession for both specialized and generalist herbivores.

In terms of robustness, the greater fragility of the initial successional stage might be caused by a reduced plant community structure and increased connectance in the network. However, this result is inconsistent with the random pattern found in all the networks, since random networks are more susceptible to extinction than a network with modulated pattern [16], [17]. We did find modules in our networks, although their arrangement did not seem to be determined by any biological factor. This also merits further study to determine whether other cluster models could better explain the observed pattern.

One important aspect of this study is that greater fragility in the interaction network, measured as reduced robustness, as well as greater connectance [27] and a lower number of compartments (which entails greater dispersion of extinctions within the network [16], [17]), were all seen in the initial succession stage. This pattern highlights the importance of the rapid establishment of tree species in recently abandoned sites in order to increase the natural regeneration of the forest and to consolidate plant-herbivore networks. It is clear that caution must be used when considering the conclusions of the indices in management decision-making [19] because ecological networks can be highly robust in the face of random removal of species, but also extremely fragile when attacks are selective, e.g. the removal of highly connected species, which are often those most abundant [46].

Exploitation of plant species by local people in Chamela is uncommon. In general, useful plant species are little appreciated by the local inhabitants [47]. However, the loss of abundant timber species such as *Apoplanesia paniculata* Persl, which is highly connected to various species of caterpillars in both mature and secondary forest, could have a large impact on the lepidopterous community. For this reason, it is suggested that the number of interactions should be considered as a criterion for protection or selection of species in restoration programs, given that the introduction of highly connected plant species [48] can conserve the properties of the system and promote the establishment of other groups of organisms [15].

Our results contrast with the conclusions of Quesada *et al.* [9] and coincide with Murphy and Lugo that proposed that the ecological succession of the TDF can reach a state of maturity very rapidly [44]. The fact that the index values that describe the fragility of the trophic networks in this study, such as those of the extinction curve, connectance and number of compartments [16], [18], [27] were no different between secondary and mature forests suggests that despite the differences in species abundance and composition across forest sucessional stages, the properties of plant-herbivore networks are quikly restored. This result is encouraging, given the large number of secondary forests in the tropics [25], [49]. One important aspect to be considered and that should be further investigated, is that the fast recovery of ecological processes in these perturbed areas has been likely influenced by their relative close distance to the main source of both plants and herbivores colonizers in the Chamela Cuixmala Biological Reserve comprising 13,142 ha of continuous forest [26]. Other studies have also shown that in tropical rainforest, when the land has been subjected to low intensity use and sources of propagules of the woody species recover rapidly [38], animal species richness can approach that of mature forests between 20 and 30 years after abandonment [43]. Recovery of ecological processes, however, is likely to be quite different if source well conserved areas are not available close to the secondary forest succession matrix.

The present study is an example of how the use of ecological networks allows us to analyze the resilience of biotic interactions in the face of environmental perturbations and to devise strategies for ecosystem conservation. We observed that the properties characterizing plant-herbivore networks can be recovered quite soon during secondary succession of the TDF. Despite differences in species richness and composition [42], the size and connectance of their networks are restored together with the recovery of the forest structure, without this affecting the specificity of the network or its susceptibility to extinction once the initial succession stage has been left behind. These results give a small glimpse into the way in which interspecific interactions occur during secondary succession and we therefore suggest that more complex studies should be carried out, considering all herbivore guilds as well as other trophic levels and species interactions, such as pollination and dispersal. Likewise, studies should be conducted to identify key species of a system, in order to carry out management and conservation actions that are appropriate to ensure the maintenance of greatest level of biodiversity within an ecosystem.

## Supporting Information

Appendix S1 Incidence of plant species in different successional stages of tropical dry forest in the Chamela region. Numbers indicate the number of Lepidopteran species found associated with that plant species. * indicates absence, 0 indicates that the plant is present in the successional stage but has no interaction with Lepidopterans.
(DOCX)

Appendix S2 Incidence of Lepidopteran species in different successional stages of tropical dry forest in the Chamela region. Numbers indicate the number of plant species where that Lepidopteran species was found. 0 indicates absence.
(DOCX)
