# Supplementary material for: Resilience in Plant-Herbivore Networks during Secondary Succession
Source: PLoS One. 2012 Dec 27;7(12):e53009. doi: 10.1371/journal.pone.0053009 (PMC3531414; doi:10.1371/journal.pone.0053009)
Supplement: Appendix S1 — Incidence of plant species in different successional stages of tropical dry forest in the Chamela region. Numbers indicate the number of Lepidopteran species found associated with that plant species. * indicates absence, 0 indicates that the plant is present in the successional stage but has no interaction with Lepidopterans. (DOCX) [file pone.0053009.s001.docx]

Appendix 1. Incidence of plant species in different successional stages of tropical dry forest in the Chamela region. Numbers indicate the number of Lepidopteran species found associated with that plant species. * indicates absence, 0 indicates that the plant is present in the successional stage but has no interaction with Lepidopterans.

|  | **Pasture** | **Early Successional** | **Late Successional** | **Mature Forest** |
| --- | --- | --- | --- | --- |
| Acanthaceae |  |  |  |  |
| Acanthaceae sp.1 | * | 4 | * | * |
| Acanthaceae sp.3 | * | * | * | 1 |
| *Justicia candicans* (Nees) L.  Benson | * | 0 | 5 | 14 |
| *Ruellia foetida* Willd. | * | * | 1 | 0 |
| Achatocarpaceae |  |  |  |  |
| *Achatocarpus gracilis* H. Walt | * | 1 | * | 7 |
| Amaranthaceae |  |  |  |  |
| *Lagrezia monosperma* (Rose)  Standl. | * | * | * | 1 |
| Anacardiaceae |  |  |  |  |
| *Spondias purpurea* L. | * | 6 | 15 | * |
| Apocynaceae |  |  |  |  |
| *Rauvolfia tetraphylla* L. | 2 | 23 | * | * |
| *Stemmadenia donnell-smithii*  (Rose ex Donn. Sm.) Woods. | * | 2 | 15 | * |
| *Thevetia ovata* (Cav.) A. DC. | * | 0 | * | * |
| Asteraceae |  |  |  |  |
| *Liabum caducifolium*  B. L. Rob. &  Bartlett | * | * | 1 | * |
| *Otopappus tequilanus* (S. Wats) B.  L. Rob. | * | * | * | 1 |
| Bignoniaceae |  |  |  |  |
| *Adenocalymma inundatum* Mart.  ex DC. | * | 2 | 0 | * |
| *Clytostoma binatum* (Thunb.)  Sandw | * | 1 | * | 2 |
| *Tabebuia chrysantha* (Jacq.)  Nicholson | * | * | 2 | * |
| *Tabebuia impetiginosa* (Mart.)  Standl | * | 1 | * | * |
| Bombacaceae |  |  |  |  |
| *Ceiba grandiflora* | * | * | * | 5 |
| Boraginaceae |  |  |  |  |
| *Bourreria purpusii* T.S. Brandg | * | 8 | 3 | 2 |
| *Cordia aff. gerascanthus* L. | * | * | 5 | 0 |
| *Cordia alliodora* (Ruiz & Pav.)  Oken | * | 5 | 3 | 1 |
| *Cordia elaeagnoides* DC. | * | * | 5 | * |
| *Cordia* sp. 1 | * | * | * | 0 |
| *Tournefortia* sp. | * | * | 0 | * |
| Burseraceae |  |  |  |  |
| *Bursera instabilis* McVaugh &  Rzed. | * | * | * | 0 |
| *Bursera* sp. 1 | * | 3 | * | 0 |
| *Bursera* sp. 2 | * | 0 | * | 3 |
| Capparaceae |  |  |  |  |
| *Capparis flexuosa* (L.) L | 1 | * | * | * |
| *Capparis indica* (L.) Druce | * | * | * | 2 |
| *Capparis verrucosa* Jacq. | * | 1 | * | 6 |
| *Crateva tapia*  L. | * | * | 8 | * |
| *Forchhammeria pallida* Liebm. | * | 1 | * | 4 |
| *Forchhammeria sessiliflora*  Standl. | * | * | 0 | 0 |
| Caricaceae |  |  |  |  |
| *Jacaratia mexicana* A. DC. | * | * | * | 2 |
| Celastraceae |  |  |  |  |
| *Hpp. Hemiangium excelsum*  (Kunth) A.C. Sm. | * | 2 | * | * |
| *Pristimera celastroides* (Kunth)  A.C. Sm. | * | * | 4 | * |
| Cochlospermaceae |  |  |  |  |
| *Cochlospermun vitifolium* (Willd.)  Spreng. | * | 1 | * | * |
| Convonvulaceae |  |  |  |  |
| *Ipomoea wolcottiana* Rose | * | 2 | * | * |
| Ebenaceae |  |  |  |  |
| *Diospyros aequoris* Standl. | 1 | 6 | * | * |
| Erythroxylaceae |  |  |  |  |
| *Erythroxylum havanense* Jacq. | * | * | * | 3 |
| *Erythroxylum rotundifolium*  Lunan | * | * | * | 1 |
| Euphorbiaceae |  |  |  |  |
| Acalypha sp. 1 | * | * | * | 1 |
| Acalypha sp. 2 | * | * | * | 5 |
| *Cnidoscolus spinosus* Lundell | * | * | 3 | * |
| *Croton alamosanus* Rose | * | * | * | 2 |
| *Croton niveus* Jacq. | * | * | * | 4 |
| *Croton pseudoniveus* Lundell | * | 5 | 2 | 12 |
| *Croton roxanae* Crozat | * | * | 15 | 28 |
| *Croton septemnervius* McVaugh | * | 0 | * | * |
| *Croton suberosus* H. B. K. | * | 6 | * | 0 |
| *Jatropha platyphylla* Muell. Arg. | * | * | 2 | * |
| *Phyllanthus botryanthus* Muell.  Arg. | * | * | * | 11 |
| *Phyllanthus mocinianus* Baill | * | * | * | 15 |
| Flacourtiaceae |  |  |  |  |
| *Casearia aculeata* Jacq. | * | 3 | * | * |
| *Casearia nitida* (L.) Jacq. | * | 28 | 24 | 5 |
| *Casearia sylvestris* Sw. | * | 4 | * | * |
| *Casearia tremula* (Griseb.)  Wright | * | 1 | 2 | 7 |
| *Prockia crucis* P. Brown ex L. | * | * | * | 5 |
| *Samyda mexicana* Rose | * | 3 | 1 | 0 |
| Hernandiaceae |  |  |  |  |
| *Gyrocarpus jatrophifolius* Domin | 4 | 9 | 4 | 3 |
| Leguminosae |  |  |  |  |
| *Acacia angustissima* (Mill.) Ktze. | 0 | * | 5 | * |
| *Acacia farnesiana* (L.) Willd. | 3 | 4 | * | * |
| *Acacia macracantha* Humb. &  Bonpl. | 4 | 8 | 3 | * |
| *Aeschynomene amorphoides* (S.  Wats) Rose ex B. L. Rob. | * | 4 | * | 0 |
| *aff. Calliandra emarginata*  (Willd.) Benth. | 0 | * | * | 7 |
| *aff. Lonchocarpus* sp. L | 10 | * | * | 0 |
| *Albizia occidentalis* Brandg. | * | * | 2 | * |
| *Albizia tomentosa* (Micheli)  Standl. | * | * | 3 | * |
| *Apoplanesia paniculata* Presl. | * | 8 | 10 | 65 |
| *Bauhinia pauletia* Pers | * | 5 | * | * |
| *Caesalpinia caladenia* Standl. | * | 19 | 27 | 7 |
| *Caesalpinia coriaria* (Jacq.) Willd. | 1 | 2 | 0 | * |
| *Caesalpinia eriostachys* Benth. | 1 | 6 | 4 | 8 |
| *Caesalpinia platyloba* S. Wats | 1 | * | 0 | 3 |
| *Calliandra emarginata* (Willd.)  Benth | * | 0 | * | * |
| *Chloroleucon mangense* (Jacq.)  Britton & Rose | * | * | * | 1 |
| *Coursetia caribaea* (Jacq.) Lavin | * | * | 1 | 13 |
| *Dalbergia congestiflora* Pitt | * | * | 4 | * |
| *Dalbergia congestiflora* Pitt | * | 9 | * | * |
| *Diphysa occidentalis* Rose. | 5 | 9 | * | * |
| *Leguminosae* sp. 8 | * | 3 | * | * |
| *Leguminosae* sp. 9 | * | 2 | 5 | * |
| *Leucaena lanceolata S. Wats* | 1 | 34 | 8 | * |
| *Lonchocarpus eriocarinalis*  Micheli | 5 | 4 | 1 | 0 |
| *Lonchocarpus guatemalensis*  Benth | * | * | 1 | * |
| *Lonchocarpus* sp. 2 | * | 7 | * | * |
| *Lonchocarpus* sp. 4 | 1 | 16 | * | * |
| *Lonchocarpus* sp. A | 1 | 7 | * | 32 |
| *Lonchocarpus* sp. F | * | * | * | 15 |
| *Lonchocarpus* sp. K | 7 | * | * | * |
| *Lonchocarpus* sp. L | 0 | 9 | * | 3 |
| *Lonchocarpus* sp. Q | 0 | * | * | * |
| *Lonchocarpus* sp. R | 2 | * | * | * |
| *Lysiloma microphyllum* Benth | * | 5 | 2 | 7 |
| *Mimosa arenosa* (Willd.) Poir.  var. *leiocarpa* (DC.) Barneby | 12 | * | * | * |
| *Mimosa* sp. 2 | 32 | * | * | * |
| *Myrospermun frutescens* Jacq. | * | * | 2 | * |
| *Piptadenia constricta* (Pers.) J. F.  Macbr. | 0 | 27 | 5 | 14 |
| *Piscidia carthagenensis* Jacq. | * | 0 | 3 | 0 |
| *Platymiscium lasiocarpum* | * | * | 5 | * |
| *Pterocarpus orbiculatus* DC. in  DC. | * | 7 | 2 | 9 |
| *Senna mollissima* (Willd.) I. & B. | * | 3 | 2 | * |
| *Senna pallida* (Vahl) I. & B. | 0 | 4 | 4 | 2 |
| *Zapoteca formosa* (Willd.) H.  Hern. | 1 | * | * | 3 |
| Malpigiaceae |  |  |  |  |
| *Bunchosia palmeri* S. Wats | * | 6 | 4 | 4 |
| *Hiraea reclinata* Jacq. | * | * | * | 1 |
| *Malphigia emilae* W. R. Anderson | 1 | 4 | 0 | 5 |
| Malvaceae |  |  |  |  |
| *Malvaceae* sp.1 | * | * | 1 | * |
| Meliaceae |  |  |  |  |
| *Trichilia trifolia* L. | * | 3 | 12 | 1 |
| Myrtaceae |  |  |  |  |
| *Psidium sartorianum* (Berg) Ndzu | * | * | * | 1 |
| Nyctaginaceae |  |  |  |  |
| *Guapira macrocarpa* Miranda | * | 6 | 14 | 12 |
| *Nyctaginaceae* sp.1 | * | * | * | 0 |
| Polygonaceae |  |  |  |  |
| *Coccoloba liebmannii* Lindau | 3 | 0 | 4 | 0 |
| *Coccoloba venosa* L. | * | 2 | * | * |
| *Podopterus mexicanus* Humb. &  Bonpl. | * | 1 | * | * |
| *Ruprechtia fusca* Fern | * | 1 | * | 6 |
| Rhamnaceae |  |  |  |  |
| *Colubrina triflora* Brongn | * | 5 | 12 | * |
| *Karwinskia latifolia* Standl. | * | * | 1 | * |
| Rubiaceae |  |  |  |  |
| *Guettarda elliptica* Sw. | * | * | 1 | 1 |
| *Hintonia latiflora* (Sessé & Moc.  ex DC.) Bullock | * | 9 | 2 | * |
| *Machaonia acuminata* Humb. &  Bonpl. | * | 8 | * | 1 |
| *Randia aculeata* L. | * | 2 | * | * |
| *Randia* sp. 1 | * | * | * | 1 |
| *Randia thurberi* S. Wats | * | * | * | 0 |
| *Rubiaceae* sp. 3 | * | * | * | 1 |
| Rutaceae |  |  |  |  |
| *Esenbeckia berlandieri* Baill | 1 | * | * | 4 |
| *Esenbeckia nesiotica* Standl | * | 1 | * | * |
| *Zanthoxyllum caribaeum* Lam. | * | * | 2 | 0 |
| *Zanthoxylum fagara* (L.) Sarg. | * | 4 | 3 | * |
| *Zanthoxylum* sp. 1 | * | 5 | 3 | * |
| *Zanthoxylum* sp. 2 | * | 1 | * | * |
| *Zanthoxylum* sp. 3 | * | * | * | 0 |
| Sapindaceae |  |  |  |  |
| *Serjania brachycarpa* A. Gray | * | * | * | 0 |
| *Thouinia paucidentata* Radlk | * | * | 7 | 22 |
| *Thouinidium decandrum* (Humb.  & Bonpl.) Radlk | * | 5 | * | * |
| Sterculiaceae |  |  |  |  |
| *Ayenia micrantha* Standl. | * | * | 10 | 12 |
| *Guazuma ulmifolia* Lam | * | * | 3 | 0 |
| Theophrastaceae |  |  |  |  |
| *Jacquinia pungens* A. Gray | 1 | 7 | 6 | 1 |
| Tiliaceae |  |  |  |  |
| *Heliocarpus pallidus* Rose | * | * | 18 | 17 |
| Ulmaceae |  |  |  |  |
| *Celtis iguanaea* (Jacq.) Sarg. | * | * | * | 0 |
| Verbenaceae |  |  |  |  |
| *Lippia mcvaughii* Moldenke | * | * | * | 1 |
