# Supplementary material for: Resilience in Plant-Herbivore Networks during Secondary Succession
Source: PLoS One. 2012 Dec 27;7(12):e53009. doi: 10.1371/journal.pone.0053009 (PMC3531414; doi:10.1371/journal.pone.0053009)
Supplement: Appendix S2 — Incidence of Lepidopteran species in different successional stages of tropical dry forest in the Chamela region. Numbers indicate the number of plant species where that Lepidopteran species was found. 0 indicates absence. (DOCX) [file pone.0053009.s002.docx]

Appendix 2. Incidence of Lepidopteran species in different successional stages of tropical dry forest in the Chamela region. Numbers indicate the number of plant species where that Lepidopteran species was found. 0 indicates absence.

|  | **Pasture** | **Early successional** | **Late successional** | **Mature forest** |
| --- | --- | --- | --- | --- |
| Arctiidae |  |  |  |  |
| *Hypercompe* sp. | 1 | 0 | 0 | 4 |
| *Lophocampa debilis* (Schaus) | 4 | 4 | 5 | 4 |
| Arctiidae sp. 1 | 1 | 5 | 3 | 0 |
| Arctiidae sp. 2 | 0 | 2 | 0 | 1 |
| Arctiidae sp. 3 | 0 | 0 | 3 | 0 |
| Arctiidae sp. 4 | 1 | 4 | 3 | 0 |
| Arctiidae sp. 5 | 0 | 0 | 0 | 1 |
| Arctiidae sp. 6 | 0 | 2 | 2 | 0 |
| Bombycidae |  |  |  |  |
| *Apatelodes palma* Druce | 0 | 0 | 0 | 2 |
| *Apatelodes pudefacta* Dyar | 0 | 7 | 4 | 3 |
| Bombycidae sp. 1 | 0 | 2 | 1 | 0 |
| Crambidae |  |  |  |  |
| *Cosmopterosis jasonhalli* Solis | 0 | 0 | 0 | 2 |
| *Dichocrocis sabatialis* | 1 | 0 | 0 | 2 |
| *Phostria citrinalis (*Druce) | 0 | 1 | 0 | 0 |
| *Phostria temira* (Stoll in Cramer & Stoll) | 0 | 4 | 2 | 1 |
| *Pilocrocis calamistis* Hampson | 0 | 0 | 1 | 4 |
| *Psara prumnides* (Druce) | 1 | 1 | 1 | 2 |
| *Syllepsis hortalis* (Walker) | 1 | 0 | 2 | 5 |
| *Symphysa lepidaria* (Stoll in Cramer & Stoll) | 0 | 0 | 1 | 0 |
| Ctenuchidae |  |  |  |  |
| *Psilopleura minax* Draudt | 0 | 2 | 1 | 0 |
| Dalceridae |  |  |  |  |
| *Dalcerides ingenita* (Edwards) | 0 | 1 | 2 | 3 |
| *Dalcerides sofia* Dyar | 0 | 2 | 0 | 1 |
| Geometridae |  |  |  |  |
| *Ametris nitocris* (Cramer) | 0 | 2 | 0 | 0 |
| Geometridae sp. 1 | 0 | 0 | 1 | 2 |
| Geometridae sp. 10 | 0 | 4 | 3 | 8 |
| Geometridae sp. 11 | 1 | 0 | 0 | 2 |
| Geometridae sp. 12 | 0 | 2 | 4 | 1 |
| Geometridae sp. 13 | 0 | 0 | 2 | 1 |
| Geometridae sp. 14 | 1 | 2 | 0 | 2 |
| Geometridae sp. 15 | 0 | 0 | 1 | 0 |
| Geometridae sp. 16 | 0 | 0 | 0 | 3 |
| Geometridae sp. 17 | 0 | 0 | 0 | 3 |
| Geometridae sp. 18 | 1 | 0 | 0 | 1 |
| Geometridae sp. 2 | 0 | 2 | 1 | 1 |
| Geometridae sp. 3 | 0 | 0 | 0 | 1 |
| Geometridae sp. 4 | 0 | 0 | 0 | 1 |
| Geometridae sp. 6 | 0 | 0 | 0 | 1 |
| Geometridae sp. 7 | 2 | 4 | 3 | 4 |
| Geometridae sp. 8 | 0 | 2 | 0 | 1 |
| Geometridae sp. 9 | 2 | 1 | 0 | 2 |
| *Glena* sp. | 0 | 0 | 0 | 2 |
| *Melanchroia vazquezae* Beutelspacher | 0 | 0 | 1 | 1 |
| *Semiothisa* sp. | 1 | 1 | 1 | 4 |
| Geometridae sp. 19 | 0 | 0 | 0 | 1 |
| Geometridae sp. 20 | 0 | 0 | 0 | 1 |
| Geometridae sp. 21 | 0 | 1 | 0 | 0 |
| Geometridae sp. 22 | 0 | 2 | 0 | 0 |
| Geometridae sp. 23 | 1 | 1 | 2 | 0 |
| Geometridae sp. 24 | 1 | 0 | 0 | 0 |
| Geometridae sp. 25 | 0 | 0 | 3 | 0 |
| Geometridae sp. 26 | 0 | 2 | 1 | 0 |
| Geometridae sp. 27 | 0 | 0 | 1 | 0 |
| Geometridae sp. 28 | 0 | 1 | 0 | 2 |
| Geometridae sp. 29 | 0 | 1 | 0 | 0 |
| Geometridae sp. 30 | 0 | 1 | 1 | 1 |
| Geometridae sp. 31 | 0 | 1 | 1 | 0 |
| Geometridae sp. 32 | 0 | 1 | 0 | 0 |
| Geometridae sp. 33 | 0 | 2 | 0 | 0 |
| Geometridae sp. 34 | 0 | 0 | 1 | 6 |
| Geometridae sp. 35 | 0 | 1 | 1 | 0 |
| Geometridae sp. 36 | 1 | 0 | 0 | 1 |
| Geometridae sp. 37 | 0 | 0 | 0 | 1 |
| Geometridae sp. 38 | 2 | 0 | 0 | 0 |
| Geometridae sp. 39 | 0 | 0 | 0 | 1 |
| Geometridae sp. 40 | 0 | 2 | 0 | 1 |
| Geometridae sp. 41 | 0 | 0 | 0 | 1 |
| Geometridae sp. 42 | 0 | 1 | 0 | 0 |
| Geometridae sp. 43 | 0 | 1 | 0 | 0 |
| Geometridae sp. 44 | 0 | 0 | 1 | 0 |
| Geometridae sp. 45 | 0 | 0 | 1 | 0 |
| Geometridae sp. 46 | 1 | 0 | 0 | 0 |
| Geometridae sp. 47 | 0 | 1 | 0 | 0 |
| Geometridae sp. 48 | 0 | 1 | 0 | 0 |
| Geometridae sp. 49 | 0 | 1 | 1 | 2 |
| Geometridae sp. 50 | 0 | 0 | 0 | 1 |
| Geometridae sp. 51 | 0 | 0 | 1 | 0 |
| Geometridae sp. 52 | 0 | 0 | 1 | 0 |
| Geometridae sp. 53 | 0 | 1 | 0 | 0 |
| Geometridae sp. 54 | 1 | 0 | 0 | 0 |
| Geometridae sp. 55 | 0 | 1 | 0 | 0 |
| Geometridae sp. 56 | 0 | 1 | 0 | 0 |
| Geometridae sp. 57 | 0 | 1 | 0 | 0 |
| Geometridae sp. 58 | 2 | 0 | 0 | 2 |
| Geometridae sp. 59 | 0 | 1 | 4 | 3 |
| Geometridae sp. 60 | 0 | 1 | 0 | 0 |
| Geometridae sp. 61 | 0 | 0 | 3 | 0 |
| Geometridae sp. 62 | 0 | 1 | 0 | 1 |
| Geometridae sp. 63 | 1 | 0 | 1 | 1 |
| Geometridae sp. 64 | 2 | 9 | 2 | 4 |
| Geometridae sp. 65 | 0 | 1 | 0 | 0 |
| Geometridae sp. 66 | 1 | 0 | 0 | 0 |
| Geometridae sp. 67 | 0 | 1 | 0 | 1 |
| Hesperiidae |  |  |  |  |
| *Astraptes gilberti* H. Freeman | 0 | 0 | 1 | 0 |
| *Eantis tamenund* (W.H. Edwards) | 0 | 0 | 1 | 0 |
| *Hesperiidae* sp. 1 | 0 | 3 | 2 | 3 |
| *Polygonus manueli manueli* Bell et Comstock | 2 | 2 | 2 | 6 |
| *Proteides mercurius* (Fabricius) | 0 | 0 | 0 | 3 |
| *Thessia jalapus* (Plötz) | 0 | 1 | 0 | 0 |
| Hesperiidae sp. 2 | 0 | 0 | 0 | 1 |
| Hesperiidae sp. 3 | 0 | 1 | 0 | 0 |
| Hesperiidae sp. 4 | 0 | 0 | 0 | 1 |
| Limacodidae |  |  |  |  |
| *Parasa* sp. | 0 | 0 | 0 | 2 |
| Limacodidae sp. 1 | 0 | 0 | 0 | 1 |
| Limacodidae sp. 2 | 0 | 1 | 0 | 2 |
| Limacodidae sp. 3 | 0 | 1 | 1 | 1 |
| Limacodidae sp. 4 | 0 | 0 | 0 | 2 |
| Lymantriidae |  |  |  |  |
| *Orgyia* sp. | 0 | 24 | 21 | 14 |
| Megalopygidae |  |  |  |  |
| *Norape tenera* Druce | 2 | 3 | 1 | 4 |
| Noctuidae |  |  |  |  |
| *Anomis editrix* (Guenée) | 0 | 2 | 4 | 8 |
| *Baniana* sp. | 0 | 2 | 0 | 0 |
| *Bleptina* sp. | 0 | 1 | 0 | 1 |
| *Codatractus* sp.1 | 0 | 0 | 0 | 1 |
| *Coxina hadenoides* Guenée | 0 | 1 | 0 | 1 |
| *Deinopa biligula* Guenée | 1 | 3 | 0 | 0 |
| *Euclystis insana* Guenee | 0 | 0 | 0 | 1 |
| *Eutelia auratrix* (Walker) | 1 | 0 | 5 | 3 |
| *Eutelia* sp. | 0 | 2 | 2 | 0 |
| *Gonodonta pyrgo* (Cramer) | 3 | 1 | 6 | 2 |
| *Isogona scindens* Walker | 1 | 0 | 0 | 0 |
| *Mimophisma* sp. | 1 | 1 | 0 | 1 |
| Noctuidae sp. 2 | 0 | 0 | 3 | 2 |
| *Phurys immunis* Guenée | 0 | 0 | 0 | 3 |
| Notodontidae |  |  |  |  |
| *Afilia venadia* Schaus | 0 | 1 | 0 | 0 |
| *Dasylophia eminens* Schaus | 1 | 5 | 3 | 2 |
| *Dicentria marimba* Schaus | 0 | 1 | 1 | 0 |
| *Hapigiodes xolotl*  Schaus | 0 | 3 | 0 | 1 |
| *Pseudhapigia brunnea* Schaus | 0 | 2 | 0 | 0 |
| *Schizura* sp. 1 | 0 | 0 | 0 | 1 |
| Notodontidae sp. 1 | 0 | 2 | 3 | 2 |
| Notodontidae sp. 2 | 0 | 3 | 3 | 0 |
| Notodontidae sp. 3 | 0 | 0 | 1 | 1 |
| Notodontidae sp. 4 | 0 | 0 | 1 | 0 |
| Notodontidae sp. 5 | 0 | 0 | 1 | 0 |
| Notodontidae sp. 6 | 0 | 0 | 0 | 2 |
| Notodontidae sp. 7 | 0 | 0 | 0 | 1 |
| Notodontidae sp. 8 | 0 | 1 | 0 | 0 |
| Notodontidae sp. 9 | 0 | 1 | 0 | 0 |
| Notodontidae sp. 10 | 1 | 2 | 0 | 0 |
| Notodontidae sp. 11 | 0 | 0 | 1 | 0 |
| Notodontidae sp. 12 | 0 | 0 | 1 | 0 |
| Notodontidae sp. 13 | 0 | 0 | 0 | 1 |
| Nymphalidae |  |  |  |  |
| *Adelpha basiloides* (Bates) | 0 | 0 | 0 | 1 |
| *Agraulis vanillae incarnata* (N. Riley) | 2 | 0 | 0 | 0 |
| *Annanea troglodyta aidea* (Guerin) | 0 | 1 | 1 | 1 |
| *Chlosyne gloriosa* Bauer | 0 | 4 | 1 | 2 |
| *Chlosyne riobalsensis* Bauer | 0 | 0 | 1 | 1 |
| *Memphis forreri* (Godman y Salvin) | 0 | 1 | 0 | 1 |
| *Memphis pithyusa* (Felder) | 0 | 1 | 0 | 0 |
| *Microtia elva elva* Bates | 0 | 1 | 0 | 0 |
| Nymphalidae sp.1 | 0 | 0 | 0 | 1 |
| Nymphalidae sp.2 | 0 | 0 | 1 | 0 |
| Nymphalidae sp.3 | 0 | 1 | 0 | 0 |
| Nymphalidae sp.4 | 0 | 1 | 0 | 0 |
| Pieridae |  |  |  |  |
| *Ascia monuste* Linnaeus | 1 | 0 | 0 | 0 |
| *Ganyra josephina josepha* (Godman & Salvin) | 0 | 2 | 0 | 4 |
| *Phoebis sennae marcellina* (Cramer) | 0 | 2 | 1 | 1 |
| *Pieriballia viardi viardi* (Boisduval) | 0 | 0 | 0 | 1 |
| Pterophoridae |  |  |  |  |
| *Hellensia chamelai* (Gielis) | 0 | 1 | 0 | 1 |
| Pyralidae |  |  |  |  |
| *Diaphania* prob. *jairulasis* | 1 | 1 | 3 | 4 |
| *Epipaschia superatalis* Clemens | 0 | 0 | 1 | 2 |
| *Pococera* sp. | 0 | 1 | 2 | 0 |
| Pyralidae sp. 1 | 0 | 0 | 0 | 0 |
| *Syllepte nr.* Cramer | 0 | 1 | 3 | 1 |
| Riodinidae |  |  |  |  |
| *Emesis emesia* Hewitson | 0 | 1 | 3 | 3 |
| Saturniidae |  |  |  |  |
| *Automeris io* Draut | 1 | 8 | 2 | 0 |
| *Hylesia continua* (Walker) | 1 | 2 | 0 | 1 |
| *Hylesia lineata* Druce | 0 | 0 | 3 | 2 |
| *Rothschildia cincta cincta* (Tepper) | 1 | 3 | 1 | 0 |
| Saturniidae sp. 1 | 0 | 1 | 0 | 1 |
| Saturniidae sp. 2 | 0 | 0 | 0 | 2 |
| Saturniidae sp. 3 | 0 | 0 | 1 | 0 |
| Saturniidae sp. 4 | 1 | 0 | 0 | 1 |
| Saturniidae sp. 5 | 0 | 1 | 0 | 0 |
| Saturniidae sp. 6 | 0 | 0 | 0 | 1 |
| Saturniidae sp. 7 | 0 | 1 | 0 | 0 |
| Sphingidae |  |  |  |  |
| *Aellopus afceculus* | 0 | 1 | 0 | 0 |
| *Erinnys crameri* (Schaus) | 1 | 0 | 1 | 0 |
| *Erinnys ello ello* (Linnaeus) | 0 | 0 | 1 | 1 |
| Sphingidae sp. 1 | 1 | 0 | 0 | 0 |
| Sphingidae sp. 2 | 0 | 0 | 1 | 0 |
| Sphingidae sp. 3 | 0 | 1 | 0 | 0 |
| Uraniidae |  |  |  |  |
| *Epiplema subapicata* (Warren) | 0 | 0 | 0 | 3 |
| Uraniidae sp. 1 | 0 | 1 | 0 | 1 |
| Urodidae |  |  |  |  |
| *Wockia chewbacca* Adamski | 0 | 5 | 2 | 1 |
| Unknown families |  |  |  |  |
| O1 | 0 | 2 | 1 | 2 |
| O100 | 0 | 0 | 0 | 1 |
| O103 | 0 | 0 | 0 | 1 |
| O105 | 0 | 0 | 0 | 1 |
| O106 | 0 | 1 | 0 | 0 |
| O108 | 0 | 1 | 0 | 0 |
| O11 | 0 | 0 | 1 | 0 |
| O110 | 0 | 2 | 0 | 0 |
| O113 | 0 | 0 | 1 | 0 |
| O115 | 0 | 0 | 1 | 0 |
| O117 | 0 | 3 | 1 | 0 |
| O119 | 0 | 1 | 1 | 0 |
| O120 | 0 | 0 | 1 | 0 |
| O124 | 0 | 1 | 0 | 0 |
| O126 | 0 | 2 | 0 | 0 |
| O127 | 0 | 1 | 0 | 1 |
| O129 | 0 | 1 | 1 | 0 |
| O13 | 0 | 0 | 1 | 0 |
| O130 | 0 | 2 | 2 | 0 |
| O134 | 0 | 0 | 2 | 0 |
| O135 | 1 | 0 | 0 | 0 |
| O136 | 1 | 0 | 0 | 0 |
| O137 | 1 | 0 | 0 | 0 |
| O138 | 1 | 0 | 0 | 0 |
| O14 | 0 | 2 | 12 | 26 |
| O140 | 1 | 0 | 0 | 0 |
| O145 | 0 | 0 | 0 | 1 |
| O146 | 0 | 2 | 1 | 2 |
| O147 | 1 | 4 | 1 | 3 |
| O148 | 0 | 0 | 0 | 2 |
| O152 | 0 | 1 | 0 | 0 |
| O153 | 0 | 1 | 1 | 1 |
| O155 | 0 | 1 | 1 | 0 |
| O156 | 0 | 0 | 1 | 0 |
| O157 | 0 | 1 | 0 | 0 |
| O158 | 0 | 1 | 1 | 1 |
| O160 | 0 | 1 | 0 | 0 |
| O163 | 0 | 0 | 0 | 1 |
| O168 | 0 | 0 | 1 | 0 |
| O171 | 1 | 1 | 0 | 1 |
| O172 | 0 | 1 | 0 | 1 |
| O174 | 0 | 0 | 0 | 1 |
| O175 | 0 | 3 | 0 | 2 |
| O176 | 0 | 0 | 0 | 1 |
| O178 | 0 | 0 | 1 | 3 |
| O179 | 0 | 0 | 0 | 1 |
| O18 | 1 | 6 | 4 | 0 |
| O180 | 0 | 0 | 1 | 0 |
| O182 | 0 | 0 | 1 | 0 |
| O184 | 0 | 1 | 0 | 0 |
| O185 | 0 | 1 | 0 | 0 |
| O188 | 0 | 1 | 0 | 0 |
| O19 | 0 | 0 | 1 | 1 |
| O190 | 3 | 10 | 2 | 0 |
| O191 | 0 | 1 | 0 | 0 |
| O193 | 0 | 1 | 0 | 0 |
| O195 | 0 | 1 | 0 | 0 |
| O196 | 2 | 0 | 0 | 0 |
| O199 | 0 | 2 | 1 | 1 |
| O20 | 0 | 0 | 1 | 1 |
| O201 | 0 | 1 | 0 | 0 |
| O206 | 1 | 0 | 0 | 0 |
| O207 | 2 | 0 | 2 | 1 |
| O21 | 1 | 1 | 0 | 1 |
| O210 | 0 | 1 | 1 | 0 |
| O211 | 0 | 4 | 1 | 1 |
| O213 | 0 | 0 | 1 | 0 |
| O214 | 0 | 0 | 2 | 1 |
| O222 | 0 | 1 | 1 | 0 |
| O224 | 1 | 0 | 0 | 0 |
| O231 | 2 | 0 | 0 | 1 |
| O233 | 0 | 0 | 0 | 1 |
| O234 | 0 | 1 | 0 | 2 |
| O238 | 0 | 0 | 1 | 0 |
| O239 | 0 | 0 | 0 | 1 |
| O240 | 0 | 0 | 0 | 1 |
| O242 | 0 | 0 | 0 | 1 |
| O248 | 0 | 0 | 0 | 1 |
| O249 | 0 | 0 | 0 | 1 |
| O250 | 0 | 0 | 0 | 1 |
| O251 | 0 | 0 | 0 | 1 |
| O252 | 0 | 0 | 0 | 1 |
| O254 | 0 | 0 | 0 | 1 |
| O255 | 0 | 0 | 0 | 1 |
| O257 | 0 | 0 | 0 | 1 |
| O258 | 0 | 0 | 1 | 4 |
| O262 | 0 | 1 | 0 | 0 |
| O263 | 0 | 1 | 0 | 0 |
| O266 | 0 | 1 | 0 | 0 |
| O268 | 0 | 0 | 1 | 0 |
| O269 | 0 | 0 | 1 | 0 |
| O270 | 0 | 0 | 1 | 0 |
| O272 | 0 | 0 | 1 | 0 |
| O273 | 0 | 0 | 1 | 0 |
| O274 | 0 | 0 | 1 | 0 |
| O276 | 0 | 0 | 1 | 0 |
| O277 | 0 | 1 | 1 | 0 |
| O278 | 0 | 1 | 0 | 0 |
| O281 | 0 | 0 | 1 | 0 |
| O283 | 0 | 0 | 2 | 0 |
| O284 | 0 | 0 | 2 | 0 |
| O287 | 0 | 1 | 0 | 4 |
| O29 | 0 | 2 | 1 | 1 |
| O290 | 0 | 1 | 0 | 3 |
| O293 | 0 | 1 | 0 | 0 |
| O298 | 0 | 0 | 0 | 1 |
| O3 | 2 | 19 | 18 | 23 |
| O302 | 0 | 0 | 0 | 2 |
| O305 | 0 | 0 | 0 | 1 |
| O309 | 0 | 1 | 0 | 2 |
| O31 | 0 | 1 | 0 | 1 |
| O310 | 0 | 0 | 0 | 1 |
| O313 | 1 | 0 | 0 | 0 |
| O316 | 2 | 1 | 0 | 0 |
| O317 | 0 | 2 | 0 | 1 |
| O319 | 0 | 1 | 0 | 1 |
| O32 | 0 | 0 | 2 | 5 |
| O322 | 0 | 0 | 0 | 1 |
| O323 | 0 | 0 | 0 | 2 |
| O328 | 0 | 1 | 0 | 0 |
| O331 | 0 | 1 | 0 | 0 |
| O332 | 0 | 1 | 0 | 0 |
| O333 | 0 | 1 | 3 | 0 |
| O337 | 0 | 1 | 0 | 0 |
| O338 | 0 | 1 | 0 | 0 |
| O340 | 0 | 1 | 1 | 1 |
| O343 | 0 | 0 | 1 | 0 |
| O350 | 0 | 1 | 0 | 0 |
| O351 | 0 | 1 | 3 | 1 |
| O356 | 0 | 3 | 0 | 0 |
| O359 | 0 | 1 | 0 | 2 |
| O360 | 2 | 0 | 0 | 0 |
| O361 | 0 | 2 | 2 | 3 |
| O363 | 0 | 1 | 0 | 0 |
| O365 | 1 | 0 | 0 | 0 |
| O366 | 1 | 0 | 0 | 0 |
| O367 | 1 | 0 | 0 | 0 |
| O368 | 1 | 0 | 0 | 0 |
| O369 | 0 | 1 | 0 | 0 |
| O371 | 0 | 1 | 0 | 1 |
| O373 | 0 | 1 | 0 | 0 |
| O381 | 0 | 0 | 1 | 0 |
| O382 | 0 | 0 | 1 | 0 |
| O383 | 0 | 0 | 1 | 0 |
| O384 | 0 | 0 | 1 | 0 |
| O385 | 0 | 0 | 1 | 0 |
| O388 | 0 | 1 | 0 | 0 |
| O39 | 0 | 0 | 0 | 1 |
| O390 | 0 | 0 | 2 | 0 |
| O391 | 1 | 1 | 0 | 0 |
| O392 | 1 | 0 | 0 | 0 |
| O393 | 0 | 0 | 0 | 1 |
| O398 | 0 | 0 | 0 | 1 |
| O4 | 0 | 1 | 0 | 1 |
| O40 | 0 | 0 | 1 | 0 |
| O406 | 0 | 0 | 0 | 1 |
| O408 | 0 | 0 | 0 | 1 |
| O409 | 0 | 0 | 1 | 1 |
| O411 | 0 | 1 | 0 | 0 |
| O413 | 0 | 0 | 0 | 1 |
| O416 | 0 | 1 | 0 | 1 |
| O417 | 0 | 0 | 1 | 2 |
| O418 | 0 | 0 | 1 | 0 |
| O419 | 0 | 1 | 0 | 1 |
| O42 | 0 | 1 | 0 | 0 |
| O420 | 0 | 1 | 0 | 0 |
| O421 | 0 | 1 | 0 | 1 |
| O422 | 0 | 1 | 0 | 0 |
| O424 | 0 | 0 | 0 | 1 |
| O426 | 0 | 0 | 0 | 1 |
| O427 | 0 | 0 | 0 | 1 |
| O429 | 0 | 0 | 0 | 1 |
| O43 | 0 | 1 | 0 | 0 |
| O430 | 0 | 0 | 0 | 1 |
| O431 | 0 | 0 | 0 | 3 |
| O432 | 0 | 0 | 0 | 1 |
| O433 | 0 | 0 | 0 | 1 |
| O434 | 0 | 0 | 0 | 1 |
| O435 | 0 | 0 | 1 | 0 |
| O436 | 0 | 1 | 0 | 0 |
| O437 | 0 | 1 | 2 | 0 |
| O438 | 0 | 0 | 1 | 0 |
| O439 | 0 | 0 | 1 | 0 |
| O440 | 0 | 0 | 1 | 0 |
| O442 | 0 | 1 | 0 | 0 |
| O443 | 0 | 0 | 1 | 0 |
| O446 | 0 | 0 | 0 | 1 |
| O447 | 0 | 0 | 0 | 1 |
| O449 | 0 | 1 | 0 | 0 |
| O451 | 0 | 1 | 1 | 0 |
| O452 | 0 | 1 | 0 | 0 |
| O454 | 0 | 1 | 0 | 0 |
| O455 | 0 | 1 | 1 | 0 |
| O459 | 0 | 0 | 2 | 0 |
| O46 | 0 | 0 | 0 | 1 |
| O463 | 0 | 0 | 0 | 2 |
| O464 | 0 | 0 | 0 | 1 |
| O465 | 0 | 0 | 1 | 0 |
| O467 | 0 | 0 | 1 | 0 |
| O468 | 0 | 0 | 1 | 0 |
| O469 | 0 | 0 | 0 | 1 |
| O47 | 0 | 0 | 0 | 2 |
| O470 | 0 | 0 | 1 | 0 |
| O471 | 0 | 0 | 1 | 0 |
| O472 | 0 | 0 | 1 | 0 |
| O474 | 0 | 0 | 0 | 1 |
| O475 | 0 | 0 | 1 | 0 |
| O476 | 0 | 0 | 1 | 0 |
| O478 | 0 | 1 | 0 | 0 |
| O479 | 0 | 0 | 0 | 1 |
| O48 | 0 | 0 | 0 | 1 |
| O480 | 0 | 0 | 1 | 0 |
| O481 | 0 | 0 | 1 | 0 |
| O483 | 0 | 0 | 0 | 1 |
| O484 | 0 | 0 | 0 | 1 |
| O485 | 0 | 0 | 1 | 0 |
| O486 | 0 | 0 | 1 | 0 |
| O487 | 0 | 0 | 0 | 1 |
| O49 | 0 | 1 | 0 | 1 |
| O5 | 0 | 1 | 0 | 1 |
| O50 | 0 | 0 | 0 | 1 |
| O501 | 0 | 1 | 0 | 0 |
| O502 | 0 | 1 | 0 | 0 |
| O504 | 0 | 0 | 0 | 1 |
| O505 | 0 | 0 | 0 | 1 |
| O506 | 0 | 0 | 0 | 1 |
| O507 | 0 | 0 | 1 | 0 |
| O509 | 0 | 1 | 1 | 0 |
| O510 | 0 | 0 | 1 | 0 |
| O512 | 0 | 0 | 1 | 0 |
| O514 | 0 | 1 | 0 | 0 |
| O516 | 0 | 0 | 1 | 0 |
| O518 | 0 | 0 | 1 | 0 |
| O522 | 0 | 1 | 0 | 0 |
| O523 | 0 | 1 | 0 | 0 |
| O524 | 0 | 1 | 0 | 0 |
| O526 | 0 | 1 | 0 | 0 |
| O527 | 0 | 1 | 0 | 0 |
| O529 | 0 | 1 | 0 | 0 |
| O53 | 1 | 1 | 1 | 0 |
| O531 | 1 | 0 | 0 | 0 |
| O532 | 0 | 0 | 0 | 1 |
| O533 | 0 | 0 | 0 | 1 |
| O54 | 0 | 2 | 2 | 4 |
| O540 | 0 | 0 | 0 | 1 |
| O541 | 1 | 0 | 0 | 0 |
| O542 | 1 | 0 | 0 | 0 |
| O543 | 0 | 1 | 0 | 0 |
| O544 | 0 | 1 | 0 | 0 |
| O545 | 0 | 1 | 0 | 0 |
| O546 | 0 | 1 | 0 | 0 |
| O548 | 0 | 0 | 0 | 1 |
| O550 | 0 | 0 | 0 | 1 |
| O551 | 0 | 0 | 1 | 0 |
| O552 | 0 | 2 | 1 | 0 |
| O556 | 0 | 0 | 1 | 0 |
| O557 | 0 | 1 | 0 | 0 |
| O559 | 0 | 0 | 1 | 0 |
| O560 | 0 | 0 | 0 | 1 |
| O561 | 0 | 0 | 0 | 1 |
| O564 | 0 | 0 | 0 | 1 |
| O565 | 0 | 0 | 0 | 1 |
| O566 | 0 | 0 | 0 | 1 |
| O6 | 0 | 1 | 0 | 1 |
| O63 | 0 | 3 | 0 | 0 |
| O64 | 1 | 2 | 0 | 0 |
| O66 | 0 | 1 | 2 | 2 |
| O67 | 0 | 1 | 4 | 7 |
| O7 | 0 | 0 | 1 | 1 |
| O72 | 0 | 1 | 1 | 0 |
| O73 | 0 | 1 | 0 | 0 |
| O74 | 0 | 2 | 3 | 2 |
| O78 | 1 | 0 | 0 | 0 |
| O82 | 0 | 0 | 1 | 0 |
| O84 | 0 | 0 | 0 | 0 |
| O85 | 0 | 0 | 0 | 1 |
| O9 | 1 | 1 | 0 | 0 |
| O91 | 1 | 0 | 0 | 1 |
| O92 | 1 | 0 | 0 | 0 |
| O94 | 1 | 0 | 0 | 0 |
| O99 | 1 | 0 | 0 | 2 |
| O192 | 1 | 1 | 0 | 0 |
| O202 | 0 | 1 | 1 | 1 |
| O204 | 0 | 2 | 0 | 0 |
| O71 | 0 | 4 | 1 | 1 |
| O88 | 1 | 0 | 0 | 0 |
